# Optical See-Through and Video See-Through Head-Mounted Displays for Percutaneous Biopsies: A Comparative Phantom Study

**DOI:** 10.1007/s00270-026-04360-3

**Published:** 2026-04-07

**Authors:** Alice M. Jacob, Alexander M. C. Böhner, Andreas Henkel, Taraneh Aziz-Safaie, Lukas Oelmeier, Nick Lenzen, Anna-Maria Odenthal, Mohammed Bahaaeldin, Aileen Schmidt, Darius Kurt, Joseph Sieber, Leon M. Bischoff, Yannik C. Layer, Dmitrij Kravchenko, Marilia Voigt, Patrick Kupczyk, Tatjana Dell, Narine Mesropyan, Alexander Isaak, Claus C. Pieper, Julian A. Luetkens, Daniel Kuetting

**Affiliations:** 1 Clinic for Diagnostic and Interventional Radiology, University Hospital Bonn, Bonn, Germany; 2 Clinic for Diagnostic and Interventional Radiology, Klinikum Stuttgart, Stuttgart, Germany

**Keywords:** Extended reality, Augmented reality, Head-mounted displays, Optical see-through, Video see-through, Apple Vision Pro, Magic Leap 2

## Abstract

**Purpose:**

To compare the added benefit of optical see-through, the Magic Leap 2 (ML2), and video see-through, the Apple Vision Pro (AVP), head-mounted displays extended reality (XR) during percutaneous biopsies on an abdominal phantom.

**Material and Methods:**

In this phantom-based prospective cohort study, sixteen radiologists (5 experienced and 11 beginners) performed six needle insertions: two without XR, two with ML2, and two with AVP. Two lesions of differing difficulty (depth and proximity to vessels) were targeted. Post-procedure CT measured accuracy (hitting the target and distance and angle to lesion center). Cognitive workload and user experience were assessed using a structured questionnaire with a rating of 0–20.

**Results:**

Beginners benefited more from XR than experienced radiologists. Beginners improved their targeting accuracy with XR, especially for the more complex lesion closer to blood vessels, where success rates increased using XR (conventional: 1/11, ML2: 2/11, AVP: 4/11), with a decrease in mean distance to target (conventional: 17.95 ± 9.14 mm; ML2: 14.21 ± 6.27 mm; AVP: 12.02 ± 7.19 mm). Advanced radiologists had overall lower success rates (conventional: 1/5, ML2: 0/5, AVP: 1/5). XR also reduced puncture time for beginner (ML2: 68.1%, AVP: 56.9% of baseline) and advanced radiologists (ML2: 79.0%, AVP: 61.7%). Questionnaire results indicated AVP was perceived as more mentally demanding, particularly by experienced radiologists (mental demand: conventional: 11, ML2: 9, AVP: 12, for beginners, and conventional: 12, ML2: 11, AVP: 17, for advanced). Beginners adapted well to both systems, showing no significant difference in perceived workload compared to conventional puncture and similar performance levels (conventional: 9, ML2: 9, AVP: 12, for beginners, and conventional: 15, ML2: 11, AVP: 7, for advanced).

**Conclusion:**

Our work demonstrates that AVP performs similar to ML2 in needle placement tasks and can be used in further research and clinical application in interventional radiology.

**Level of Evidence:**

No level of evidence.

**Graphical Abstract:**

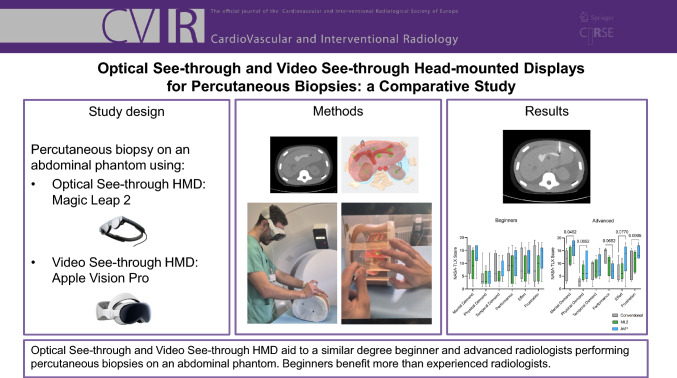

**Supplementary Information:**

The online version contains supplementary material available at 10.1007/s00270-026-04360-3.

## Introduction

Percutaneous biopsies are essential in oncology for obtaining tissue samples to confirm cancer diagnosis and guide appropriate therapy. These procedures are often image-guided, typically using ultrasound, computed tomography (CT), or cone-beam CT. While generally safe, CT-guided biopsies can be challenging depending on lesion location and patient anatomy. They require radiologists to interpret 2D CT planes and mentally reconstruct 3D anatomy, a process that demands expertise [1, 2]. In addition, reliance on ionizing radiation highlights the need for methods that reduce exposure [3].

Extended reality (XR) technologies have increasingly been explored for medical applications. XR encompasses a spectrum of immersive technologies, including virtual reality (VR), augmented reality (AR), and mixed reality (MR) [4, 5]. These modalities can be accessed via head-mounted displays (HMDs) or through mobile devices such as tablets and smartphones. HMDs can be broadly categorized based on their method of integrating the real world. Optical see-through (OST) HMDs—common in AR systems—allow users to view the real environment directly through transparent lenses, with digital content superimposed [6]. Examples include the Magic Leap 2 (ML2) and Microsoft HoloLens. Video see-through (VST) HMDs—typical of MR systems—digitally reconstruct the user’s surroundings using cameras, creating a mediated view of reality [6]. Examples include the Apple Vision Pro (AVP) and Meta Quest. In the context of image-guided interventions, OST devices have been extensively evaluated in phantom and cadaver studies. These studies report positive outcomes such as reduced procedure and planning time, decreased radiation exposure during CT-guided procedures, and increased operator accuracy [7–10]. In contrast, the use of VST HMDs in CT-guided needle placement remains less investigated.

In this study, we present a direct comparison of VST and OST HMDs for CT-guided procedures. Both types of device have different characteristics that could be useful during a puncture procedure, yet direct comparison on clinical scenarios is lacking. Our purpose is to compare the two device types for tasks and functions necessary during a puncture. Specifically, we evaluate the registration accuracy and stability of the Magic Leap 2 (an OST device, Magic Leap, Inc., Plantation, FL, USA) and Apple Vision Pro (a VST device, Apple Inc., Cupertino, CA, USA) during a 2D alignment task and during CT-guided punctures on an abdominal phantom. The registration test evaluates the intrinsic performance of each HMD on a task where the clinical skill differences between participants do not play a confounding role. The phantom biopsy test allows for a clinically relevant scenario to be evaluated, with the specific challenges a CT-guided procedure entails.

## Materials and Methods

### Head-Mounted Displays

We evaluated the ML2 (Magic Leap, Inc., Plantation, FL, USA) as an OST device and the AVP (Apple Inc., Cupertino, CA, USA) as a VST device (Supplementary Fig. 1). The ML2 runs on Android software and the AVP on VisionOS.

The prospective study focused on testing the differences between the ML2 and the AVP in two different stages. First, we performed registration tests to evaluate the intrinsic capacity of the HMDs. In this test, no specific skill from the operator was necessary. The second test with the abdominal phantom provides help elucidating the use of the HMDs in a closer to clinical scenario. Because this test requires specific clinical skills that are acquired during residency training, it is relevant to compare the performance of professionals at different experience levels.

### Registration Tests

**Fig. 1:**
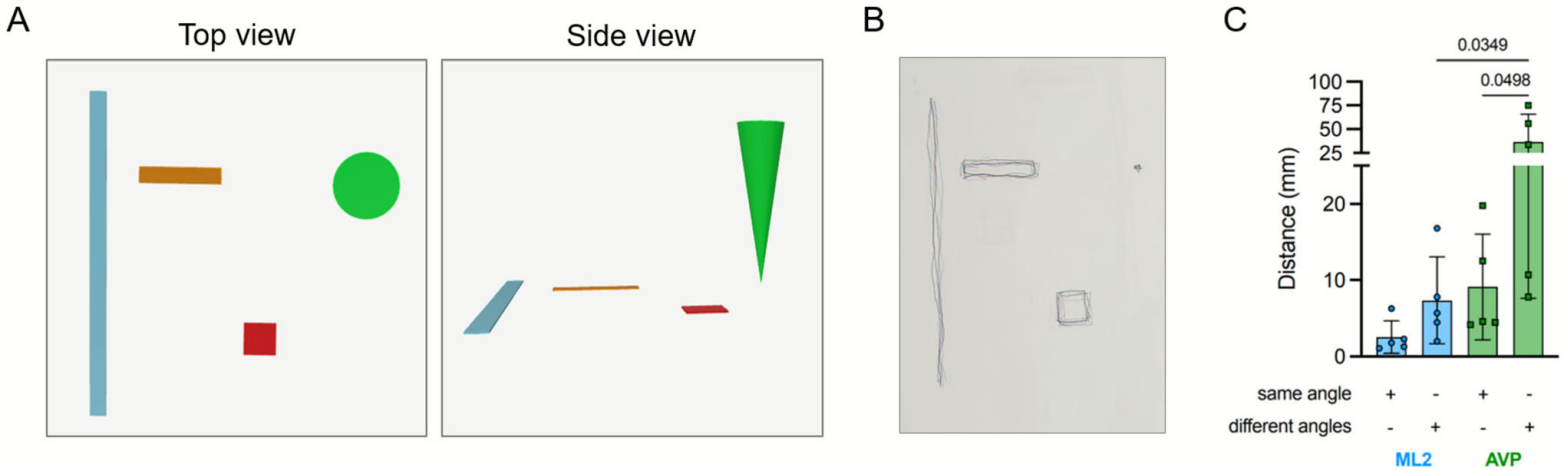
2D registration task for the ML2 and AVP. **A** Model created with blender and rendered on the ML2 and AVP. **B** Example of the task with the ML2. Each color line represents a different viewing direction. The distance between the lines was measured and plotted in C. **C** Distance of the different tests with the HMDs, n = 5 participants. Results shown as mean and standard deviation, two-way ANOVA. ML2, Magic Leap 2, AVP, Apple Vision Pro

A holographic model consisting of a cube, a rectangle, a cone, and a line was created in Blender, an open-source 3D creation software, and exported as.gbl and.usdz for the ML2 and AVP, respectively (Fig. 1A). The model was projected on a plain A4 sheet and the users drew the edges of each element from three different angles two times, making a total of 6 tests (Fig. 1B-C). To adopt the different viewing angles, the user walked around a table where the models were projected. The angles were 90° (perpendicular) and 130° (diagonal) from the first position (0°). We assessed positional repeatability across time (same viewing angle—first drawing compared to second drawing from the each position) and across different angles (paired distances between drawings made from 0°, 90°, and 130°), by measuring the distance between pairs of drawings of the different elements. Five participants (one scientist and four residents) performed the task (AMJ, AMCB, AH, TAS, and LO).

### Phantom Biopsies

**Fig. 2:**
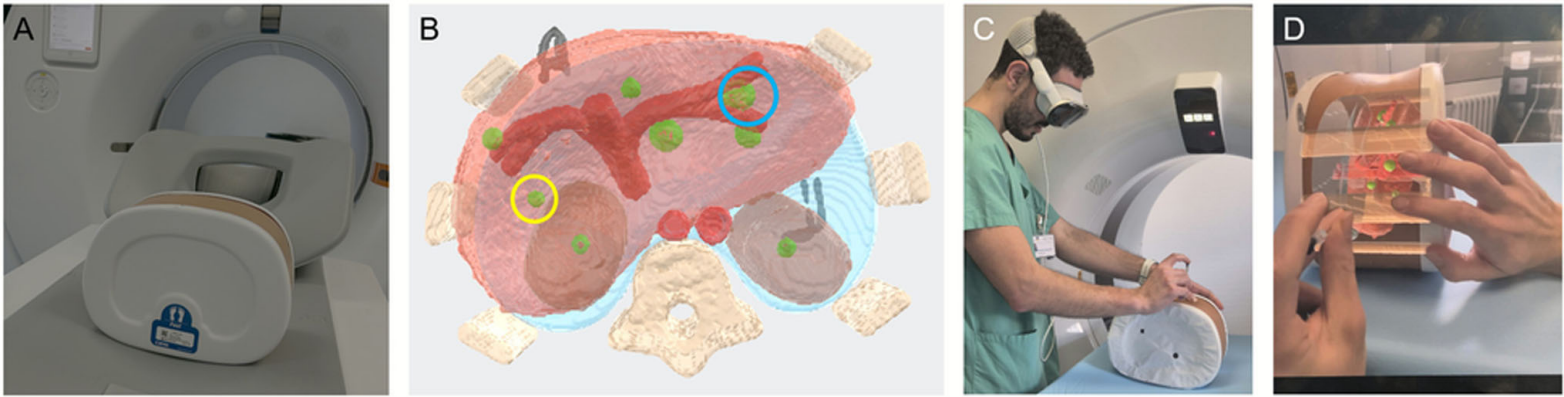
Hologram of the phantom was overlaid on the physical phantom using the HMDs. **A** View of the phantom. **B** 3D model used for the ML2 and the AVP. Target lesions used on the study, yellow circle represents Lesion 1 and blue circle represents Lesion 2. **C** Puncture procedure using the AVP. **D** View from the AVP during puncture. ML2, Magic Leap 2, AVP, Apple Vision Pro

An abdominal phantom (Triple Modality 3D Abdominal Phantom, Sun Nuclear, Melbourne, Australia) containing six hepatic lesions and one renal lesion per kidney was used (Fig. 2A). Models projected in the HMDs were segmented from CT based on density values using Fiji and rendered using Blender (Fig. 2B). The models were generated to display the organs with different colors and were given different alpha blend values (to adjust transparency). The following organs were displayed: bones (beige, alpha = 0.5), kidneys (brown, alpha = 0.4), liver (light red, alpha = 0.4), lung (light blue, alpha = 0.3), blood vessels (dark red, alpha = 0.7), and the lesions (green, alpha = 0.7). The same settings were used for both devices. Two liver lesions were chosen as target for the biopsies (Fig. 2B), with varying levels of difficulty: Lesion 1 (65 mm deep, 5 mm diameter, isolated) and Lesion 2 (40 mm deep, 14 mm diameter, adjacent to vessels).

Radiologists with different degrees of experience in CT-guided procedures (AMCB, AH, TAS, NL, AO, MB, AS, DKur, JS, LMB, YCL, DKr, MV, PK, TD, DKue) performed six punctures: two conventional, two with ML2, and two with AVP (Fig. 2C-D). Radiologist were recruited based on experience. The beginner group comprised residents that infrequently perform CT-guided procedures during their clinical work. Advanced radiologists are fellow and attending clinicians who frequently (> 50 year) perform CT-guided punctures. Participant’s level of familiarity with XR was interrogated and categorized in four levels: no experience; use less than one time per month; use less than one time per week; and use several times per week. Order in which each group of punctures (with HMD or conventional) was performed was randomized for each participant. Participants were given an introduction about each HMD and sufficient time to learn the skills necessary to perform the punctures. The introduction included the necessary hand and eye setups, manipulation of the models with the controllers (for the ML2) and with hand movements (for the AVP), and overlaying the model with the abdominal phantom. No practice punctures were performed. Training lasted until participants felt comfortable to start the tasks. There was no additional training for the conventional puncture, aside from the clinical training each participant received during their clinical education. After training and performing the punctures with each device, participants rated sufficiency of preparation with a 7-point Likert scale where 1 was “The preparation did not suffice at all” and 7 was “The preparation was absolutely sufficient.”

Each trial consisted in one attempt to reach each target, with no chance to reposition the needle. Needle approach was left to each participant criteria. Following each system, participants completed a NASA Task Load Index questionnaire (NASA-TLX) [11]. The NASA-TLX is a standardized test used in deployment of new technologies that evaluates the following parameters: mental demand of performing the task, physical demand, temporal demand, performance and success perception, effort necessary, and frustration level, on a scale of 1 to 20.

**Fig. 3:**
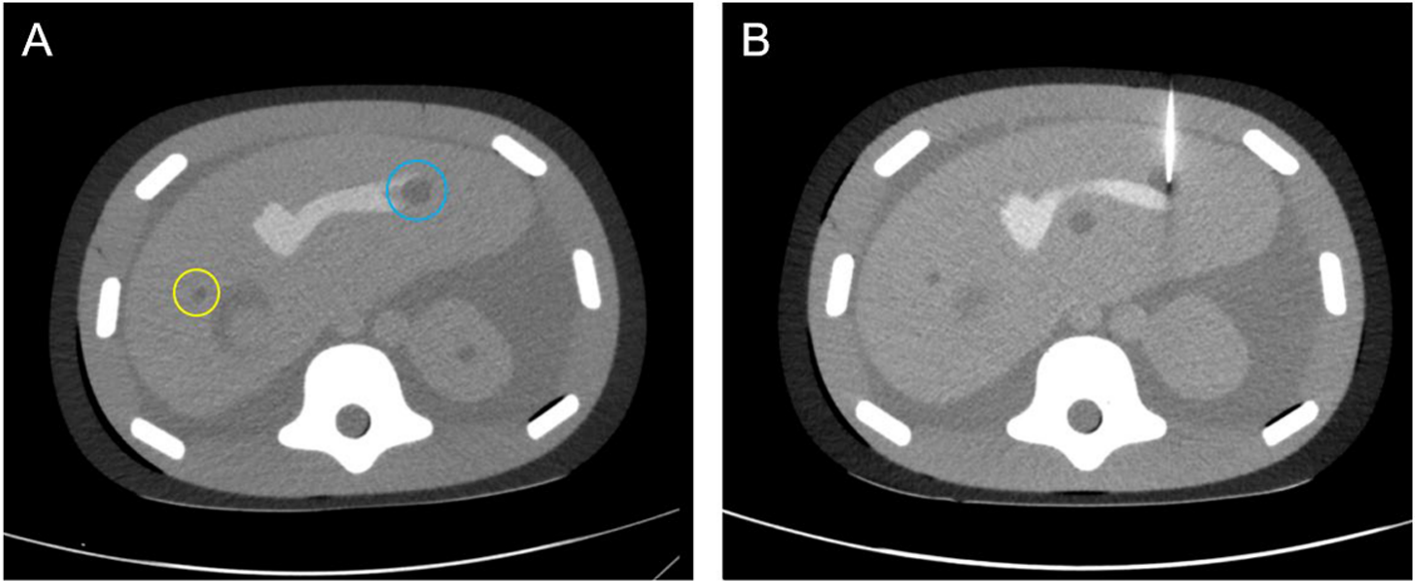
CT of the abdominal phantom used for the punctures. **A** CT with bone window of the phantom. Target lesions used on the study, yellow circle represents lesion 1 and blue circle represents lesion 2. **B** CT with bone window of an example of lesion 2 being hit with the needle

Post-procedure CT scans were used to determine performance, measuring the minimum 3D distance (in mm) from lesion center to the tip of the needle and needle angle as a 3D vector from the entry point of the needle to the center of the lesion in mm (Fig. 3). Measurements were taken on a blind manner by AMJ.

Time to perform the punctures was recorded. Time to prepare for each puncture was not included. Preparation time means time needed to evaluate the CT and measuring the necessary distances on the conventional puncture and, for the HMDs, the time needed to align the model to the phantom.

### Statistical Evaluation

Statistical evaluation and graph representation were carried out with SPSS (version 28.0) and GraphPad Prism (version 10.4.0). Registration and puncture metrics were analyzed with two-way ANOVA. NASA-TLX was evaluated by two-way ANOVA with Tukey’s post hoc correction. Continuous variables are reported as mean ± SD and ordinal/discrete variables as median (IQR).

## Results

### Intrinsic HMD Registration

Positional repeatability assessment of the AVP showed a mean deviation of 9.1 ± 6.9 mm from the same angle and, 36.7 ± 29.0 mm across angles (*p* = 0.01, Fig. 1C). For the ML2, deviation was 2.6 ± 2.1 mm from the same angle and 7.4 ± 5.7 mm across angles (*p* = 0.62). When comparing between devices, repeatability from the same angle was similar (AVP: 9.1 ± 6.9 mm vs. ML2: 2.6 ± 2.1 mm, *p* = 0.51), but the AVP demonstrated greater variability when comparing across angles (AVP: 36.7 ± 29.0 mm vs. ML2: 7.4 ± 5.7 mm, *p* = 0.008). Results are summarized in Supplementary Table 1.

### Phantom Biopsies

Sixteen radiologists (5 experienced and 11 beginners) performed a set of six punctures each (two conventional, two with ML2, and two with AVP). Mean experience with CT-guided procedures of the beginner group was 2.2 ± 1.3 years and of the advanced group was 7.4 ± 2.5 years (*p* = 0.0003). Mean age of the beginner group was of 28.5 ± 1.9 and of the advanced groups was 37.2 ± 3.6 (*p* = 0.0002). Male participants comprised 72.7% (8/11) of beginners and 60% (3/5) of advanced radiologists (*p* > 0.99). Within the beginner group, 2/11 participants had prior experience with XR using at least one HMD system several times per week. Within the experience group, 1/5 participants had prior contact with XR, using at least one HMD less than 1 time per week. All other participants (9/11 beginners and 4/5 experienced) were naïve to HMDs. Learning time with the HMDs was rated as 5.5 (5–7) for ML2 and 6 (5–7) for AVP.

**Fig. 4:**
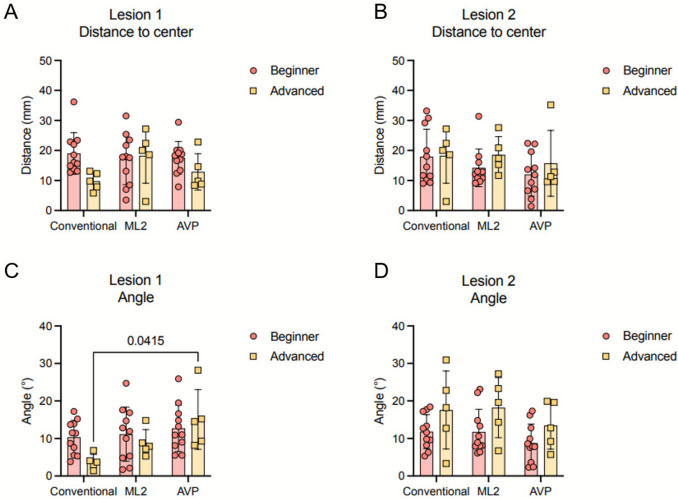
Distance of the tip of needle to the center of target lesion for the punctures with and without the HMDs. **A** Lesion 1. **B** Lesion 2. Angle offset of the needle to the center of target lesion for the punctures with and without the HMDs. **C** Lesion 1. **D** Lesion 2. Data represented as mean and standard deviation and analyzed with a two-way ANOVA. ML2, Magic Leap 2, AVP, Apple Vision Pro

Experienced radiologists performed better than beginners for lesion 1, based on the lower mean distance to lesion and angle of needle, even though not significant (Fig. 4A–D). For lesion 2, performance without XR did not differ between groups (Fig. 4B, D). The use of XR for the puncture of lesion 1 did not provide any benefit in terms of distance to lesion and angle (Fig. 4A, C). In fact, the rate of successful hits for this lesion did not change with the use of either headset by beginner and advanced radiologists (Table 1). For lesion 2, XR did not affect advanced radiologists but increased successful attempts for beginners (Table 1). Even though not significant, the distance to the center of lesion for beginners was also decreased with the use of ML2 and AVP (conventional: 17.95 ± 9.14 mm, ML2: 14.21 ± 6.27 mm, AVP: 12.02 ± 7.19 mm) (Fig. 4B). Results for all the punctures are summarized in Supplementary Table 2. Overall, the time required to perform punctures was reduced, with the effect being more pronounced among less experienced radiologists. Beginners using the ML2 required only 68.1 ± 24.0% of the time compared to conventional puncture (*p* < 0.0001, conventional: 125.4 ± 51.1 s, ML2: 88.2 ± 47.4 s), while those using the AVP needed just 56.9 ± 28.3% of the time (*p* = 0.0001, AVP: 63.3 ± 27.3 s). Advanced users wearing the ML2 needed 79.0 ± 18.8% (*p* = 0.0007, conventional: 90.8 ± 42.4 s, ML2: 75.2 ± 51.6 s) and the AVP 60.7 ± 25.8% (*p* = 0.006, AVP: 59.0 ± 50.1 s) of the time of the conventional puncture.

**Table 1:** Successful punctures with each headset

Lesion	Group	Conventional	ML2	AVP
1	Beginner	0/11	1/11	0/11
	Advanced	1/5	0/5	1/5
2	Beginner	1/11	2/11	4/11
	Advanced	1/5	0/5	1/5

ML2, Magic Leap 2; AVP, Apple Vision Pro. Number of successful attempts/Total number of attempts per group. Successful attempts are defined as hitting the target lesion

**Fig. 5:**
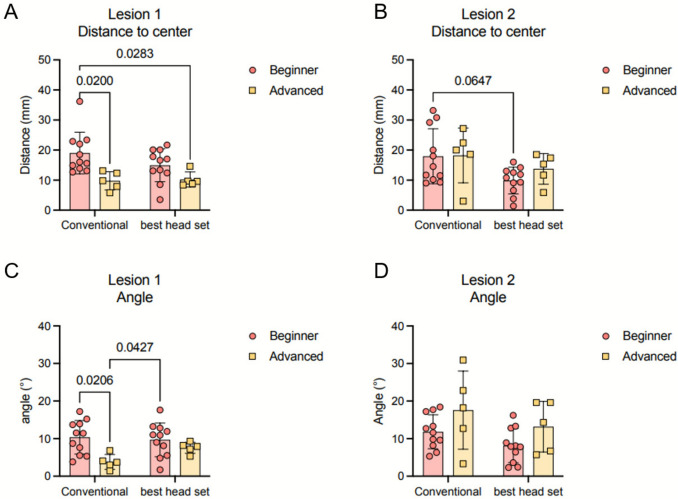
Distance of the tip of needle to the center of target lesion for the punctures with and without the best HMD. **A** Lesion 1. **B** Lesion 2. Angle offset of the needle to the center of target lesion for the punctures with and without the best HMD. **C** Lesion 1. **D** Lesion 2. Data represented as mean and standard deviation and analyzed with a two-way ANOVA

Most participants performed better with one of the HMDs but less effectively with the other, relative to conventional puncture, based on distance to lesion, angle, and hit rate. No specific pattern on performance was noticed. Overall, 5 out of 11 beginner radiologists and 3 out of 5 advanced radiologists achieved better performance with the ML2. When considering only the results obtained with each participant’s best-performing system, the beneficial effect of using XR during punctures becomes more evident among less experienced radiologists compared to their more advanced counterparts (Fig. 5, Supplementary Table 3).

**Fig. 6:**
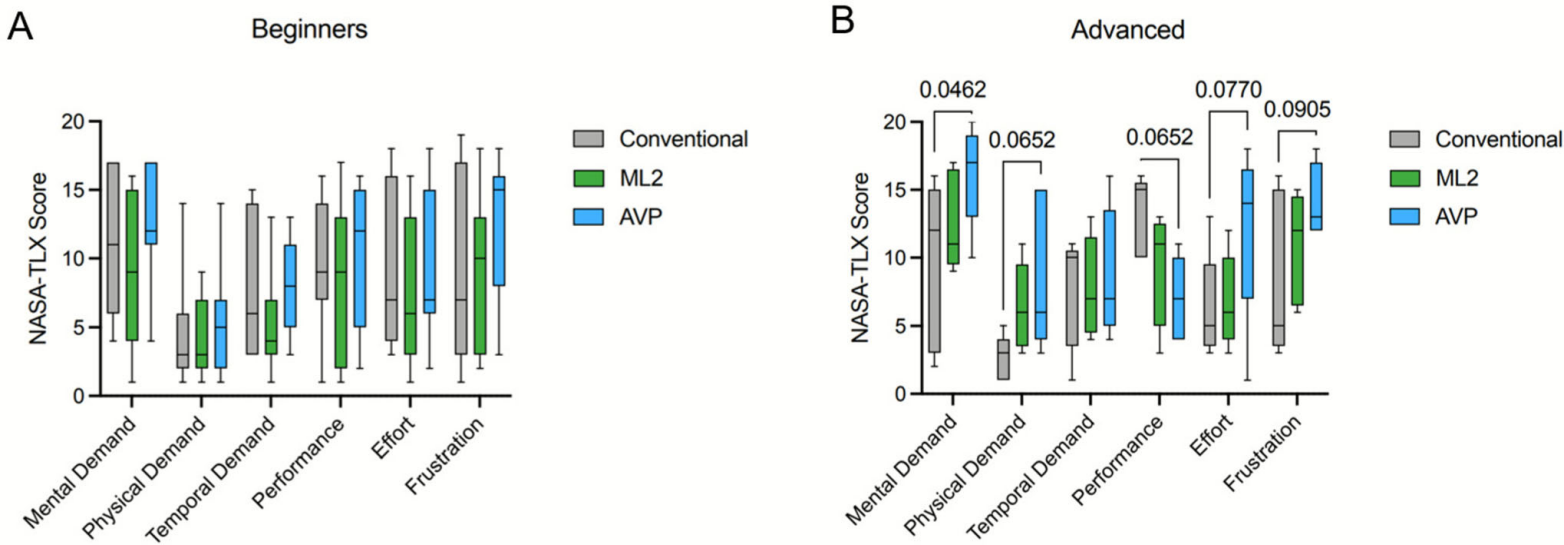
Mental and physical load evaluation of the ML2 and AVP. **A** NASA-TLX scores given by the beginner radiologist after the punctures. **B** NASA-TLX scores given by the advanced radiologist after the punctures. Data plotted as a Box and Whiskers graph, with 5–95 percentile. Statistical evaluation with a two-way ANOVA with Tukey’s multiple comparison test. ML2, Magic Leap 2, AVP, Apple Vision Pro

Beginner radiologists demonstrated better adaptability to the new technologies and the HMDs, showing no differences in workload between the conventional method and either headset (Fig. 6A, Supplementary Table 4). When comparing the two HMDs, the AVP tended to be associated with higher mental, physical, and temporal demand, as well as increased frustration (Fig. 6). Despite these higher workload scores, the AVP was also associated with a higher performance.

In contrast, advanced radiologists found the AVP to be more mentally demanding than the conventional method (*p* = 0.05) (Fig. 6B). Physical and temporal demands, along with effort and frustration, also tended to be higher with the AVP, which corresponded to a decrease in performance. Despite these differences, 8/11 beginners and 3/5 advanced radiologists preferred the ML2.

## Discussion

CT-guided procedures are routine in radiology, but present challenges related to patient motion and anatomy [1, 2]. A key factor is the radiologist’s 3D awareness of anatomy, since enhanced spatial perception can improve safety and diagnostic yield. XR technologies have been proposed to optimize this process [3].

HMDs fall into two categories: optical see-through (OST) or video see-through (VST). Comparing the performance of an OST device (for example the Magic Leap 2, ML2) and a VST device (for example the Apple Vision Pro, AVP), particularly for medical applications is therefore relevant. Effective use of XR devices in clinical procedures depends on precise and stable registration of holograms within the real-world environment. In the current study, the ML2 demonstrated greater spatial stability compared to the AVP in a 2D drawing task. In terms of accuracy, the ML2 performed similarly to what has been reported for the Microsoft HoloLens 1 and 2, showing navigation errors of up to 4 mm in phantom studies [12–16]. VST systems have been less extensively studied for this application. One report using the HTC Vive headset—a VST device—demonstrated an accuracy of 3.5 mm and a repeatability of 2.5 mm [17], exceeding the AVP’s performance in our study.

OST HMDs have been thoroughly evaluated for puncture procedures in phantom models and cadavers, with the Microsoft HoloLens being the most commonly employed system [7–10]. Previous studies indicate that less experienced radiologists, particularly younger residents, derive greater benefit from XR guidance during punctures compared to their more advanced counterparts [7]. These findings are consistent with our own observations. While less experienced users generally demonstrated improved performance with at least one of the HMDs—either the ML2 or the AVP—this effect was less apparent among experienced radiologists. The added benefit for less experienced radiologist might be related with the improved spatial awareness experienced with XR. More experienced radiologists have had longer time to develop this skill and, therefore, could benefit less from the HMDs. Other potential interfering factors are different levels of tech savviness between the groups and or different levels of expertise in video games [18].

More advanced users also tended to assign less favorable NASA-TLX scores to the HMDs, especially to the AVP, reporting higher levels of mental and physical demand compared to less experienced participants. This could be due to the greater experience that they already have puncturing effectively without HMD support, therefore feeling less comfortable with changes to an already effective protocol. A similar study with US- and XR-guided needle placements suggests a similar effect, where experienced radiologists slightly decreased in accuracy with XR, but non-radiologists improved significantly [19]. Furthermore, advanced radiologists were also older than beginners, which might contribute to a lower acceptance of new technologies. The observation that younger radiologists benefit more from XR-based guidance further underscores the potential of these systems as valuable educational tools. Future studies should investigate the teaching impact of XR in CT-guided procedures, with particular emphasis on comparing pre- and post-XR performance in conventional puncture tasks.

A greater benefit of both the ML2 and the AVP was observed for the more technically challenging lesion, which was difficult to target due to its proximity to blood vessels. An increased number of beginners were able to successfully reach this lesion when using HMDs, and a reduction in the distance to the lesion center was also noted. These findings further highlight the potential of XR technologies in enhancing spatial understanding in complex anatomical scenarios.

Despite earlier differences in 2D accuracy between ML2 and AVP, no differences were seen in puncture performance. Neither system proved consistently superior. Preferences, however, favored the ML2, aligning with NASA-TLX results where the AVP scored higher in mental, temporal, and physical demand and frustration. Users also commented on heaviness of the AVP and a tendency to dry the eyes.

The results of this study demonstrate a clear preference for ML2 over AVP for clinical applications, particularly for puncture procedures. The existing body of literature—extensive not only for the ML2 but also for the Microsoft HoloLens—further supports the use and integration of OST devices in clinical settings. By design, VST systems tend to be heavier, which may have contributed to the lower usability ratings of the AVP and to the overall preference for the ML2 among participants.

To date, no comparable studies evaluating the AVP in puncture or needle placement procedures have been identified, limiting the ability to contextualize its performance within the broader VST landscape. As a relatively recent addition to the market, scientific investigations into the AVP’s application in the medical field remain limited. However, recent reports have begun to explore its potential use as a more ergonomic alternative to traditional monitors during laparoscopic procedures [20–22].

Our work has some limitations that must be considered. The number of punctures performed by each participant with each system is relatively low. Other studies request that participants target all lesions on a phantom in each condition (with and without HMD) [7, 8]. A similar approach would dilute any bias or luck effect that might have taken place. Our single-puncture setup enabled controlled comparison between the different punctures, but differed from typical clinical scenarios, which may have disadvantaged more experienced radiologists. Although Phantom studies can be very elucidative, they cannot directly be translated to human application. One of the large difficulties present in CT-guided procedures is patient motion, for example, due to breathing or peristaltic movements. Application of HMDs during biopsy for procedures in regions that are highly subjected to intrinsic patient motion will have to take the said motion in consideration. Despite the limitations, our work provides a valuable comparison between two types of HMD systems on a medical task, helping define situations where each type of XR and HMD might be incorporated in clinical routine.

## Conclusion

This work contributes to the growing body of evidence supporting the use of HMDs in clinical applications and the education of clinicians. Here, OST and VST systems were directly compared during biopsy procedures on an abdominal phantom, with both user performance and preference systematically recorded. The use of HMDs was found to improve needle placement by enabling faster and more accurate punctures for most operators. User preferences tended to favor the ML2 over the AVP, primarily due to the greater comfort and ease of use reported.

## Supplementary Information

Below is the link to the electronic supplementary material.

Supplementary Material 1

## Data Availability

Post-procedure CT scans and model.gbl and.usdz files will be facilitated by the authors upon reasonable request.
