# Supplementary material for: Optical See-Through and Video See-Through Head-Mounted Displays for Percutaneous Biopsies: A Comparative Phantom Study
Source: Cardiovasc Intervent Radiol. 2026 Apr 7;49(5):970–9. doi: 10.1007/s00270-026-04360-3 (PMC13156115; doi:10.1007/s00270-026-04360-3)
Supplement: Supplementary file 1 — Supplementary Material 1 [file 270_2026_4360_MOESM1_ESM.docx]

**Optical See-through and Video See-through Head-mounted Displays for Percutaneous Biopsies: a Comparative Phantom Study**

Alice M. Jacob^1^, Alexander M.C. Böhner^1^, Andreas Henkel^1^, Taraneh Aziz-Safaie^1^, Lukas Oelmeier^1^, Nick Lenzen^1^, Anna-Maria Odenthal^1^, Mohammed Bahaaeldin^1^, Aileen Schmidt^1^, Darius Kurt^1^, Joseph Sieber^1^, Leon M. Bischoff^1^, Yannik C. Layer^1^, Dmitrij Kravchenko^1^, Marilia Voigt^1^, Patrick Kupczyk^1^, Tatjana Dell^1^, Narine Mesropyan^1^, Alexander Isaak^1^, Claus C. Pieper^1^, Julian A. Luetkens^1^, Daniel Kuetting^1,2^

Clinic for Diagnostic and Interventional Radiology, University Hospital Bonn, Bonn, Germany

Clinic for Diagnostic and Interventional Radiology, Klinikum Stuttgart, Stuttgart, Germany

Correspondence:

Daniel Kuetting

[d.kuetting@klinikum-stuttgart.de](mailto:d.kuetting@klinikum-stuttgart.de)

**Supplementary Figure 1**


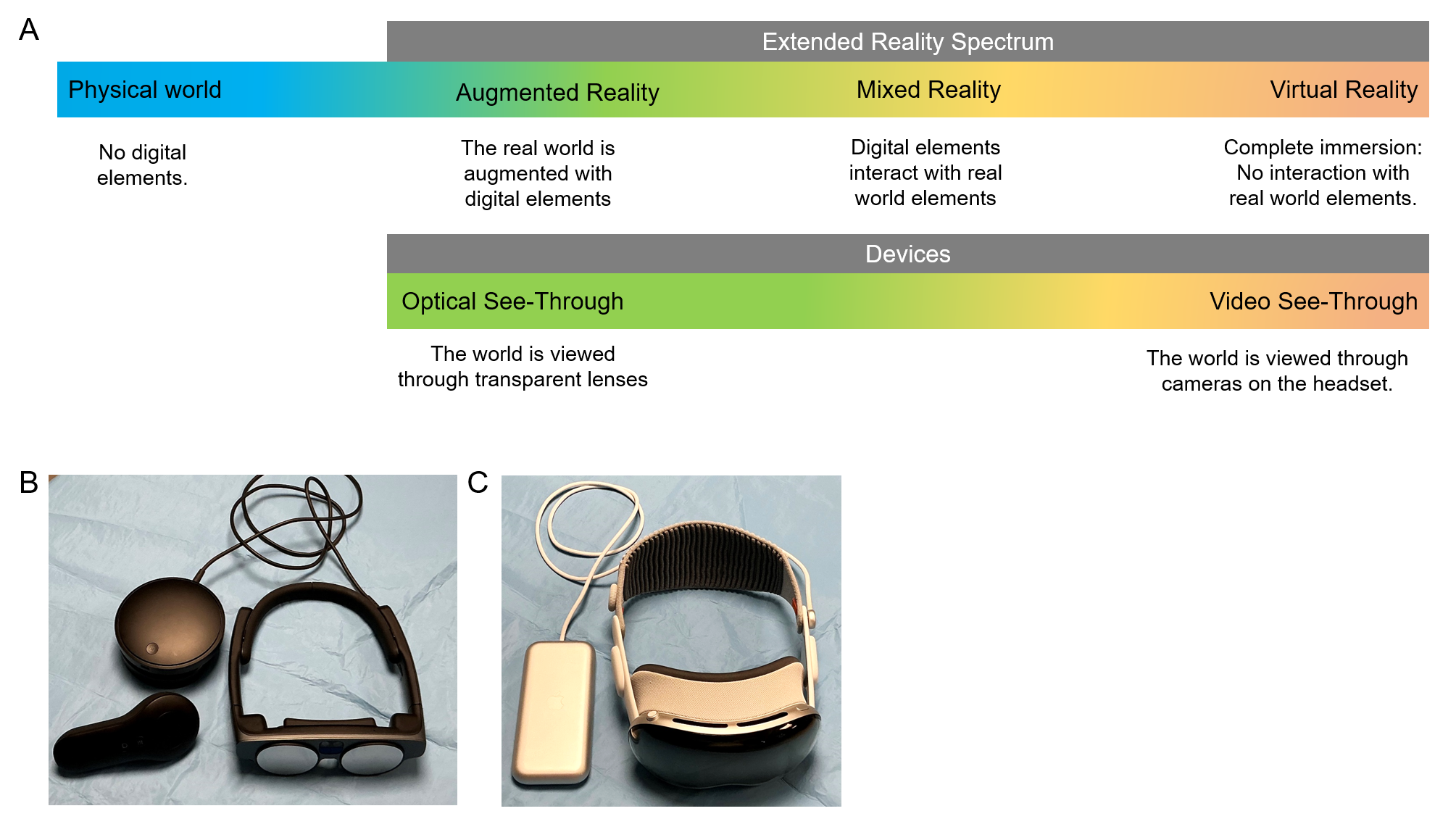


**Supplementary Figure 1:** (A) The extended reality spectrum includes Augmented Reality, Mixed Reality, and Virtual Reality. In Augmented Reality, the real word is complemented with digital elements projected through the headsets. In Mixed Reality, digital elements can interact with real world elements. In Virtual Reality, there is a complete immersion with the virtual world. To experience Extended Reality, there is a need for specific devices, mainly in the form of headsets. Optical See-Through Devices allow the user to visualize the real world environment through transparent lenses. An example of these devices are the Magic Leap 2 (B). Video See-Through Devices utilize cameras to record the real world environment and project it as video through the lenses. An example of these devices are the Apple Vision Pro (C).

**Supplementary Table 1: HMD‘s registration**

|  | Same angle | | Different angle | |  |
| --- | --- | --- | --- | --- | --- |
|  | Mean distance (mm) | SD (mm) | Mean distance (mm) | SD (mm) | p-value^1^ |
| ML2 | 2.6 | 2.1 | 7.4 | 5.7 | 0.9581 |
| AVP | 9.1 | 6.9 | 36.7 | 29.0 | 0.0498 |
| p-value^2^ | 0.9025 | | 0.0349 | |  |

Two-way ANOVA with Tukey’s post-hoc correction for multiple comparisons. ^1^ p-value for the comparison of different angles with the same headset, ^2^ p-value for the comparison of different systems from the same angle ML2, Magic Leap 2; AVP, Apple Vision Pro; SD, Standard Deviation.

**Supplementary Table 2: Quantification of distance and angle deviation from center of lesion with and without HMDs during punctures on an abdominal phantom**

|  | Beginner | | | Advanced | | |
| --- | --- | --- | --- | --- | --- | --- |
|  | Conv. | ML2 | AVP | Conv. | ML2 | AVP |
| **Lesion1** |  | | | | | |
| Distance (mm) | 19.0  ± 6.9 | 17.1  ± 8.5 | 17.6  ± 5.4 | 9.78  ± 3.0 | 18.2  ± 9.1 | 12.9  ± 6.1 |
| Angle (°) | 10.3  ± 4.5 | 11.1  ± 7.2 | 12.7  ± 6.2 | 3.80  ± 2.0 | 8.80  ± 3.6 | 15.1  ± 8.0 |
| **Lesion2** |  | | | | | |
| Distance (mm) | 17.9  ± 9.1 | 14.2  ± 6.3 | 12.0  ± 7.2 | 18.2  ± 9.1 | 18.6  ± 6.1 | 15.7  ± 11.0 |
| Angle (°) | 11.8  ± 4.5 | 11.8  ± 6.0 | 8.76  ± 5.1 | 17.6  ± 10.4 | 18.2  ± 8.0 | 13.4  ± 6.3 |

Beginner and advanced radiologists performed two needle placements with and without HMDs. Data shown as mean ± SD. Conv., Conventional method; ML2, Magic Leap 2; AVP, Apple Vision Pro; SD, Standard Deviation, CvsM, pair comparison between conventional method and ML2, CvsA pair comparison between conventional method and AVP, MvsA pair comparison between ML2 and AVP

**Supplementary Table 3: Quantification of distance and angle deviation from center of lesion with and without each participant’s best HMD during punctures on an abdominal phantom**

|  | Beginner | | Advanced | |
| --- | --- | --- | --- | --- |
|  | Conv. | Best HMD | Conv. | Best HMD |
| **Lesion1** |  | | | |
| Distance (mm) | 19.0  ± 6.9 | 14.9  ± 5.5 | 9.78  ± 3.0 | 10.2  ± 2.5 |
| Angle (°) | 10.3  ± 4.5 | 9.68  ± 4.5 | 3.80  ± 2.0 | 7.58  ± 1.5 |
| **Lesion2** |  | | | |
| Distance (mm) | 17.9  ± 9.1 | 9.87  ± 4.5 | 18.2  ± 9.1 | 13.8  ± 5.1 |
| Angle (°) | 11.8  ± 4.5 | 8.15  ± 4.5 | 17.6  ± 10.4 | 13.2  ± 6.7 |

Beginner and advanced radiologists performed two needle placements with and without HMDs. Data shown as mean ± SD. Conv., Conventional method; HMD, Head-mounted Display; SD, Standard Deviation, Conv. Vs Best HMD, pair comparison between conventional method and participant’s best HMD.

**Supplementary Table 4: NASA-TLX reports after puncture with and without HMDs**

|  | Beginners | | | Advanced | | |
| --- | --- | --- | --- | --- | --- | --- |
|  | Conv. | ML2 | AVP | Conv. | ML2 | AVP |
| Mental Demand | 11 (6-17) | 9 (4-15) | 12 (11-17) | 12 (3-15) | 11 (9.5-16.5) | 17 (13-19) |
| Physical Demand | 3 (2-6) | 3 (2-7) | 5 (2-7) | 3 (1-4) | 6 (3.5-9.5) | 6 (4-15) |
| Temporal Demand | 6 (3-14) | 4 (3-7) | 8 (5-11) | 10 (3.5-10.5) | 7 (4.5-11.5) | 7 (5-13.5) |
| Performance | 9 (7-14) | 9 (2-13) | 12 (5-15) | 15 (10-15.5) | 11 (5-12.5) | 7 (4-10) |
| Effort | 7 (4-16) | 6 (3-13) | 7 (6-15) | 5 (3.5-9.5) | 6 (4-10) | 14 (7-16.5) |
| Frustration | 7 (3-17) | 10 (3-13) | 15 (8-16) | 5 (3.5-15) | 12 (6.5-14.5) | 13 (12-17) |

Beginner and advanced radiologists performed two needle placements with and without HMDs and responded to a standardized questionnaire consisting of the NASA Task Load Index. Results shown as median and Interquartile Range. Conv., Conventional method; ML2, Magic Leap 2; AVP, Apple Vision Pro.
